# Management of drug supply chain information based on “artificial intelligence + vendor managed inventory” in China: perspective based on a case study

**DOI:** 10.3389/fphar.2024.1373642

**Published:** 2024-07-16

**Authors:** Jianwen Shen, Fengjiao Bu, Zhengqiang Ye, Min Zhang, Qin Ma, Jingchao Yan, Taomin Huang

**Affiliations:** ^1^ Department of Pharmacy, Eye & ENT Hospital, Fudan University, Shanghai, China; ^2^ Information Center, Eye & ENT Hospital, Fudan University, Shanghai, China

**Keywords:** artificial intelligence (AI), vendor managed inventory (VMI), supply processing and distribution (SPD), enterprise resource planning (ERP) system, drug supply chain

## Abstract

**Objectives:**

To employ a drug supply chain information system to optimize drug management practices, reducing costs and improving efficiency in financial and asset management.

**Methods:**

A digital artificial intelligence + vendor managed inventory (AI+VMI)-based system for drug supply chain information management in hospitals has been established. The system enables digitalization and intelligentization of purchasing plans, reconciliations, and consumption settlements while generating purchase, sales, inventory reports as well as various query reports. The indicators for evaluating the effectiveness before and after project implementation encompass drug loss reporting, inventory discrepancies, inter-hospital medication retrieval frequency, drug expenditure, and cloud pharmacy service utilization.

**Results:**

The successful implementation of this system has reduced the hospital inventory rate to approximately 20% and decreased the average annual inventory error rate from 0.425‰ to 0.025‰, significantly boosting drug supply chain efficiency by 42.4%. It has also minimized errors in drug application, allocation, and distribution while increasing adverse reaction reports. Drug management across multiple hospital districts has been standardized, leading to improved access to medicines and enhanced patient satisfaction.

**Conclusion:**

The AI+VMI system improves drug supply chain management by ensuring security, reducing costs, enhancing efficiency and safety of drug management, and elevating the professional competence and service level of pharmaceutical personnel.

## 1 Introduction

With the aging population, advancements in medical technology, emergence of new treatments and drugs, and widespread use of specialized medications, public healthcare expenditure will continue to rise. Healthcare expenditure is rapidly increasing at an annual growth rate of nearly 20% (Yuan et al., 2021). In order to address high medical costs for the general public and excessively high drug prices in China, the zero-markup drug policy was implemented in 2017 for hospital sales. This reform promotes the separation of medicine provision from healthcare services (Zhou et al., 2023). Eliminating markups on drugs has led to changes in revenue structure for public hospitals, with drug procurement expenses becoming a significant component of their expenditures (Deng et al., 2018). Simultaneously, in order to optimize drug expenditure and regulate drug circulation, China has implemented the National Centralized Drug Purchase (NCDP) since 2018 (Zhang et al., 2023). However, challenges have been identified in the implementation of national centralized procurement by medical institutions, including the absence of a scientific estimation system resulting in significant disparities between purchase volume and utilization within these institutions. This discrepancy has led to shortages or even unmet demand for certain drugs, thereby impacting their clinical usage (Gong et al., 2021). Therefore, optimizing and innovating new supply chain models represents an effective measure towards ensuring enhanced drug supply.

Furthermore, increased tasks such as medication wastage management and maintenance costs for drug storage have added pressure to daily operations management, resulting in a continuous increase in operating costs. It is widely believed that supply chain costs can make up 40%–50% of healthcare institutions’ expenses (Chen et al., 2013), suggesting that improving supply chain performance could be crucial for enhancing operational efficiency and reducing costs (Pal et al., 2021).

However, hospital supply chains have unique characteristics that set them apart from typical industrial supply chains. Many hospitals have tried different business management concepts to enhance their supply chains and apply them to healthcare facilities (Amerine et al., 2017; da Silva and de Mattos, 2019). While some have succeeded, others have failed, resulting in significant losses (Agarwal and Garg, 2012; Lesego et al., 2023). In recent years, the rapid development of information technology has enabled the application of digital intelligent supply chain systems in hospital drug supply guarantee systems, thanks to new technologies like big data, artificial intelligence, industrial internet, and blockchain.

Some scholars attribute this to the relative complexity of healthcare supply chains, including robust national and international regulatory frameworks, challenges in demand forecasting due to patient heterogeneity, and limited knowledge of the supply chain among key decision-makers such as pharmacists and physicians involved in drug procurement (Bialas et al., 2023).

The Eye and ENT Hospital of Fudan University is a world-renowned hospital and research center specializing in ophthalmology and otolaryngology, integrating medical care, education, and research to provide comprehensive patient care for the health of the eye, ear, nose, throat, head and neck. According to the China Hospital Specialist Reputation Ranking List, our otorhinolaryngology department has proudly held the #1 ranking for 14 consecutive years while our ophthalmology department ranks among the top 3 nationally (top 1 in the Yangtze River Delta). Led by academicians from Chinese Academy of Sciences as well as various world-class outstanding professors and scientists, we are dedicated to advancing our understanding of diseases affecting these regions through cutting-edge research. In the past decade, our business has expanded from 2 to 4 campuses across the city, with 894 beds now available. Medical insurance drug expenses have grown at an annual rate of approximately 10%. Currently, our hospital has over 1700 staff members. The hospital manages more than 2 million outpatient clinic and emergency department visits annually, along with performing over 120,000 surgical procedures each year. Leveraging digital transformation as a foundation, our hospital has developed a sophisticated ‘smart hospital operation management’ system that explores the realms of operational informatization, intelligent management, and medical digitization. We continuously delve into and analyze the specific needs of hospitals to expedite the realization of data-driven intelligent decision-making management, thereby effectively enhancing the hospital’s level of refinement and work efficiency. To meet the urgent need for a cost-effective operational model that facilitates standardized management across all campuses, our hospital established a multidisciplinary development team comprising experts from supplier companies as well as hospital leaders, information technology application developers, pharmaceutical specialists, and financial experts. In July 2014, we initiated construction of a digital drug supply chain system platform. In the process of digital transformation, hospitals will encounter significant challenges. The implementation of a digital supply chain can incur substantial costs, necessitating considerable investments in technology, training, and infrastructure. Ensuring compliance with privacy regulations also poses a challenge within the digital supply chain. As our hospital undergoes internal and supply chain-wide digital transformations, there is an increased sharing of critical data across the supply chain, leading to a surge in sensitive data volume and escalating cyber risks. Moreover, successful implementation of the digital supply chain requires individuals with relevant technical expertise and management knowledge. Through software and hardware upgrades and nearly 10 years of exploration and practice, we have gradually realized a digitized supply chain management model for drugs in our hospital.

The case report outlines the methods and results of developing an innovative digital drug supply chain platform in our hospital. By comparing performance before and after implementation, this report provides valuable insights into multi-campus drug management experiences that can inform decision-making for hospital drug supply chain management.

## 2 Literature review

The supply chain is a logistics network comprising suppliers, manufacturers, warehouses, distribution centers, and channel providers. With the advent of Industry 4.0, emerging technologies such as the Internet, digital technology, and artificial intelligence (AI) can enhance supply chain operations by leveraging extensive data analysis to offer actionable insights. This facilitates improved inventory evaluation and strengthens supply chain resilience (Zamani et al., 2022), thereby generating novel economic and business value (Nunez-Merino et al., 2020).

Artificial intelligence refers to machine-based intelligence compared to natural human or animal intelligence. In current supply chain management practices, AI primarily operates in single-task scenarios such as predictive inventory management as well as data analysis and processing tasks (Katuu, 2021). It enhances logistics automation and information flow while fostering collaboration among suppliers, service providers, and customers (Wamba-Taguimdje et al., 2020).

Intelligent supply chains are customer-centric, integrated systems that operate on a global scale and leverage advanced technologies to provide valuable products or make affordable services more accessible (Schallmo et al., 2019; Seyedghorban et al., 2020). Business leaders harness these new technologies to gain a competitive advantage while enhancing financial performance and adding value (Witkowski, 2016). By implementing a digital supply chain strategy, managers can anticipate an annual efficiency increase of 4.1% alongside revenue growth of 2.9% per year (Büyüközkan and Göçer, 2018).

The Enterprise Resource Planning system (ERP) is an integrated management information system designed to enhance operational efficiency and harmony within enterprises, thereby improving overall enterprise quality. It incorporates multiple modules that facilitate cross-institutional information integration (Katuu, 2021). It includes finance, sales, procurement, production and so on. ERP system can help enterprises manage business processes, improve production efficiency, reduce costs and so on. The development of the ERP system has evolved through various stages including single systems, multi-systems, open systems, cloud systems, and artificial intelligence systems. The concept of ERP originated in the 1940s and took shape during the 1970s and 1980s primarily as a material planning system aimed at assisting companies in inventory calculation and procurement needs. By the early 1990s, ERP shifted its focus towards breaking down information silos by fully integrating different departments and business processes (Kogetsidis et al., 2008). In the early 2000s, ERP gradually embraced internet and mobile technologies with cloud-based solutions becoming mainstream (Bjelland and Haddara, 2018). During the 2010s, intelligence and digitalization emerged as prominent themes driving ERP development.

These emerging supply chain technologies and enterprise planning management methods are gradually being implemented in the healthcare sector. In healthcare, efficient supply chain management programs empower organizations to enhance quality, reduce costs, and optimize performance (Elmuti et al., 2013). Healthcare institutions require pragmatic approaches to achieve operational efficiency and cost reduction while upholding safety standards (Uthayakumar and Priyan, 2013; Novais et al., 2019). Drug shortages persist across numerous countries (Bochenek et al., 2018; Shukar et al., 2021). Advanced and intelligent supply chain management systems can swiftly acquire, analyze, and predict drug shortage information while also mitigating or preventing such shortages to a certain extent. Therefore, bolstering supply chain management is imperative for enhancing the operational capabilities of healthcare organizations (George and Elrashid, 2023).

Undoubtedly, the healthcare industry exhibits significant complexity and specificity, distinguishing it from perishable products, manufacturing, and other industrial production sectors. Consequently, its logistics costs are elevated, directly impacting public health and safety. The pharmaceutical supply chain is subject to various influencing factors such as inventory demand and supply dynamics, characteristics of healthcare inventory items and storage facilities, distribution systems for inventory management, replenishment policies, growth in service levels, patient medical conditions, physician prescribing behavior, as well as interactions among involved parties (Saha and Ray, 2019).

Recently, extensive research has been conducted by scholars on drug supply chain management in the healthcare industry (Table 1). Several studies have established conceptual frameworks for assessing the pharmaceutical supply chain and identifying risk factors within it. Wang et al. proposed a framework for managing the drug supply chain, which identified the main types of uncertainty and risk involved (Wang and Jie, 2020). Botes et al. developed a new conceptual framework that enables decision-makers and supply chain managers to assess the pharmaceutical supply chain and identify favorable opportunities for public-private integration (Botes et al., 2022). In order to reduce costs in the drug supply chain, numerous researchers have explored optimization models for drug inventory management. Franco suggested building a simulation model to evaluate the cost of drug supply chains in Colombian hospitals, providing pricing and reimbursement decisions for the medical insurance sector (Franco, 2020). Additionally, two distinct mathematical models were proposed to incorporate empirical data and simulated scenarios for evaluating pharmacy-hospital systems under demand uncertainty, while considering stochastic factors associated with demand, cost, and drug delivery times. This approach enables the determination of optimal policies that can reduce current hospital supply and administration costs by 16%, while also identifying an acceptable expiration date that minimizes the overall wastage of drugs (Franco and Alfonso-Lizarazo, 2020).

**TABLE 1:** Relevant Literature review of pharmaceutical supply chain research in the healthcare industry since 2020.

	Research category			Research area			Research object						
	CTR	MM	OCS	PI	DS	HP	SCC	IM	CM	QD	LD	OE	PA
Wang and Jie (2020)	√			√			√						
Zandkarimkhani et al. (2020)		√	√			√		√	√	√	√		
Franco and Alfonso-Lizarazo (2020)		√				√	√	√	√				
Franco (2020)		√				√			√				
Papalexi et al. (2020)			√			√						√	
Goodarzian et al. (2021a)		√		√	√	√		√			√		
Goodarzian et al. (2021b)		√		√	√				√		√		
Goodarzian et al. (2022)		√	√								√		
Botes et al. (2022)	√				√							√	
Elarbi et al. (2022)		√	√			√			√				
Tucker and Daskin (2022)		√	√	√	√			√					
George and Elrashid (2023)		√	√			√		√		√		√	
Jebbor et al. (2023)			√			√		√					
Shashi (2023)		√	√	√								√	
Current research			√		√	√		√	√	√	√	√	√

CTR, conceptual theory research; MM, mathematical model; OCS, original case study; PI, pharmaceutical industry; DS, drug supplier; HP, hospital pharmacy; SCC, supply chain characteristics; IM, inventory management; CM, cost management; QD, quantity demand; LD, logistics distribution; OE, operational efficiency; PA, pharmacy administration.

Goodarzian et al. proposed a novel mixed integer nonlinear programming model to address the production-distribution-inventory-order-routing problem in multi-objective drug supply chain networks, aiming to reduce both the total cost and delivery time of drugs (Goodarzian et al., 2021a). Additionally, they designed sustainable drug supply chain management networks that consider ecological factors in pharmacies and hospitals, with the objective of minimizing environmental impact (Goodarzian et al., 2021b). Furthermore, they developed a comprehensive mixed integer linear programming model for the current network, considering multiple periods, products, objectives, and echelons. Additionally, they proposed three hybrid meta-heuristic algorithms to design sustainable resilient healthcare networks (Goodarzian et al., 2022). Elarbi et al. established a mathematical model aimed at reducing the overall supply chain cost. Their findings revealed that information sharing led to an average cost savings exceeding 14% (Elarbi et al., 2022). Zandkarimkhani et al. designed a drug supply chain network model and validated it through a case study conducted in Tehran/Iran (Zandkarimkhani et al., 2020). Tucker and Daskin proposed a related model focusing on drug supply chain configuration, disruption risk assessment, and recovery speed prediction for anticipating drug shortages (Tucker and Daskin, 2022).

In addition, expert interviews were conducted by researchers to address drug supply chain issues. Papalexi et al. conducted interviews with 22 hospital and community pharmacy pharmacists in the United Kingdom and Greece, collected and analyzed data, and concluded that finance, communication, waste, and complexity pose challenges within the supply chain (Papalexi et al., 2020). George and Elrashid investigated the relationship between inventory levels, demand forecasting, and management performance through questionnaires distributed to private hospital pharmacies (George and Elrashid, 2023). Jebbor et al. developed a comprehensive automated inventory replenishment system that seamlessly integrates with hospital business operations (Jebbor et al., 2023). Additionally, structured interviews were conducted with supply chain managers of pharmaceutical companies in the United States to explore their strategies for digitizing integrated supply chain ecosystems (Shashi, 2023). These studies analyze factors influencing supply chain efficiency, inventory forecasting, and other related aspects. Although there are limited empirical studies describing measures to enhance internal hospital supply chains along with their effects, recent research primarily focuses on model design rather than quantitative research. Furthermore, most of these studies originate from scientific research institutions rather than specific sectors within the supply chain itself. From the perspective of the hospital’s drug management department, this study analyzes strategies for optimizing the drug supply chain, explores the impact of implementing these optimization plans on enhancing pharmaceutical care in hospitals, and provides quantitative descriptions to support further research in this area.

## 3 Methods

### 3.1 Establishment of project construction team

The entire system comprises supplier, pharmacy, and finance divisions. The implementation of the business system necessitates continuous optimization of operational processes and interdepartmental cooperation with the support of hospital-level resources, taking into full consideration the requirements of both pharmaceutical management and financial systems. The drug intelligent supply chain construction team consists of hospital project leaders, pharmacists, financial personnel, information engineers, and enterprise supply chain experts. Pharmacists are responsible for designing drug procurement, supply management, inventory control functions among others. Financial staff oversee functional design related to drug billing reconciliation. Information department technical staff and enterprise supply chain technical experts are accountable for function realization. Project leaders coordinate various departments to achieve data standardization while resolving issues associated with independent systems and information silos.

### 3.2 The establishment of system framework

We have collaborated with our suppliers to establish a drug supply chain system. The overall business architecture consists four components:

(1) Supply/Processing/Distribution System (SPD System) for hospital drug procurement, inventory management, drug distribution, and reporting.
(2) Order Management System (OMS) for tracking relocated drugs’ delivery status, acceptance confirmation, and receipt confirmation;
(3) VMI for reconciliation and settlement invoices.

The entire platform integrates intelligent functions like automatic generation of purchasing plan, order management, distribution, inventory control, and financial reconciliation. Supplementary Figure S1 illustrates the layout of this system framework.

(4) Supplier’s Enterprise Resource Planning (ERP) systems.

Currently, our hospital has successfully integrated the drug ordering system, logistics distribution information, invoice pricing data, and other relevant information into the supplier’s ERP system. This integration enables effective monitoring and timely reminders for the supply chain and logistics department regarding transportation progress, inventory levels, as well as demand and supply dynamics. Additionally, suppliers can periodically review their inventory status post-consumption through the ERP system.

### 3.3 The establishment of intelligent SPD system

The aim is to enable medical professionals to focus on their core responsibilities through logistics outsourcing, thereby reducing labor costs, improving supply management efficiency, and enhancing core competitiveness (Lee et al., 2018). This approach also contributes to mitigating medical accidents (Chen et al., 2022). Our hospital has applied this concept and developed the following functional modules in the SPD pharmaceutical service system:

SPD System (Version 1.5, China National Pharmaceutical Group Corporation LTD) interfaces with suppliers’ Enterprise Resource Planning (ERP) systems and the government’s Medical Procurement Supervision Information System, allowing efficient distribution of goods and ensuring accuracy in drug procurement. It also integrates with the Hospital’s internal drug Information System (HIS, version 5.00.1.0158, Shanghai United Networks and Information Co., Ltd), automatically retrieving and managing comprehensive drug information for efficient pharmaceutical management services.

#### 3.3.1 Supply

Procurement and supply management for suppliers involves automated generation of purchase and replenishment plans, which are subsequently uploaded to the supplier side of the system platform after review. Upon receiving an order, the supplier simultaneously generates delivery documents and settlement documents, facilitating smooth delivery processes.

#### 3.3.2 Processing

Management of first-level logistics warehouse operations within the hospital includes drug warehouse acceptance procedures where pharmacists can utilize handheld devices to compare procurement information, batch numbers, prices, etc. Medicines are assigned to specific hospital codes upon storage while inventory management can also be conducted through handheld devices.

#### 3.3.3 Distribution

Involves transferring drugs from the pharmacy to secondary warehouses such as outpatient pharmacies or inpatient pharmacies. Based on application requirements from these secondary warehouses, drugs are dispatched accordingly using the SPD management system for activities like acceptance, warehousing, inventory control, consumption tracking, and application processing.

The System uses big data analysis for automatically generate purchasing plans, improving efficiency and stability in drug supply at hospitals. It facilitates seamless drug supply and transportation within and between hospitals, improving patient access to medical care and convenience. To ensure interoperability of drugs and accurate accounting, a two-step confirmation process is implemented in inventory management, along with added functionality to track drug transportation. In the monthly inventory, the system can self-check whether there are drugs in transit, such as one party out of the warehouse, and the other party is not in the warehouse, the system will automatically intercept the operation of the inventory report generation. Additionally, the system supports querying detailed sales information for specific drugs to view complete stock flow during a certain period and monitor drug consumption.

### 3.4 The establishment of VMI platform

**FIGURE 1:**
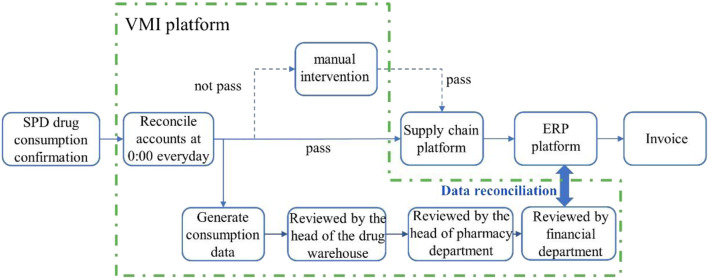
AI-based automation and resolution process. The consumption data generated by the HIS terminal and SPD is automatically reconciled daily, with the reconciliation results synchronized to the HIS storage and ERP terminals for invoice generation. The invoices from the ERP terminal are uploaded to the platform, which then transmits them back to the HIS end as a basis for hospital payment settlement.

It is crucial to enhance the resilience of the supply chain and avoid disruptions (Rehman and Ali, 2022). Rapid and effective response to changes in drug supply and demand is essential (Ozdemir et al., 2022). Several researchers have investigated the management of inventory, inventory turnover rate, reduction in inventory levels and costs by suppliers (Riewpaiboon et al., 2015; Atnafu and Balda, 2018; Salas-Navarro et al., 2024). Currently, this approach has been adopted by numerous Chinese enterprises such as Volkswagen and SGM (Wang et al., 2008). However, due to the intricacies involved in drug management, its application within the pharmaceutical supply chain remains limited. To improve management efficiency, reduce total costs of drug procurement, distribution and inventory, and minimize hospital drug inventory levels, our hospital has introduced VMI mode into drug supply chain management since February 2019. VMI is a cost-optimized inventory control method where suppliers can access real-time inventory data and consumption patterns to replenish stock based on actual demand models and trends (Mohamadi et al., 2024). We have integrated the SPD System, Procurement Platform, Internal HIS, and Financial Management System in our hospital to establish a VMI platform (version 1.0, China National Pharmaceutical Group). Based on artificial intelligence, the automated reconciliation process is illustrated in Figure 1.

To implement the VMI model in hospital drug management, it is imperative to establish a continuous collaboration strategy between hospitals and suppliers, wherein the supplier assumes responsibility for inventory management instead of following the traditional approach of separate inventory management. The hospital oversees agreement implementation and makes necessary adjustments to drive ongoing enhancements in inventory management and efficiency. Following VMI implementation, there should be a shift from conventional freight invoice methods to post-use settlement mode, settling accounts based on actual monthly consumption. This approach not only avoids tying up hospital funds but also ensures accurate settlement amounts. Moreover, suppliers only need to issue invoices once a week without requiring additional deliveries or invoicing, thereby reducing both supplier and hospital settlement management workload. The VMI platform consists of three major business management platforms: order management, reconciliation management, and invoice management.

#### 3.4.1 Intelligent drug order management

Following manual audit in the SPD system, purchase data is automatically transmitted to the OMS platform. The OMS platform then retrieves drug delivery status information. Once warehouse staff confirms receipt of the shipment within the OMS, drug data is subsequently relayed back to the warehouse module of the SPD system.

#### 3.4.2 Drug reconciliation management

This module automatically reconciles the daily drug revenue data from the hospital’s HIS system with the drug consumption data from SPD System, as shown in VMI reconciliation interface in Supplementary Figure S2.

#### 3.4.3 Drug invoice management

The main functions of this module include supplier invoice data uploading, automatic invoice matching, error invoice handling, invoice statistics and queries, SPD inventory inquiries, as well as sales and inventory tracking. When the drug price is adjusted, the system facilitates manual reconciliation.

#### 3.4.4 Pharmaceutical inventory and sales report

The integration of pharmaceuticals and finance is the organic fusion of pharmaceutical management and financial management within an organization, promoting the development of pharmaceutical administration.

After completing the drug reconciliation process, our hospital’s SPD System can intelligently generate supplier inventory reports, as well as internal logistics inventory reports for multiple hospital regions and departments. The logistics reconciliation within a hospital reflects the relationship between the pharmacy, outpatient pharmacy, and inpatient pharmacy in that specific area, as shown in Supplementary Table S1. The automatic reconciliation function for drug allocation between different areas of the hospital facilitates the calculation of total drug outflow from Department A in Area A to Department B in Area B during a specified period. This total amount will be equivalent to the total drug inflow received by Department B from various departments in Area A, and *vice versa* using the same principle. The intelligent reconciliation of drugs between hospital campus can be seen in Supplementary Table S2. The automatic reconciliation reports within and between areas provide detailed information on departmental initial balances, final balances, stock movements, and inter-departmental transfers of drugs. This achieves precise management of pharmaceutical accounting throughout the entire hospital while ensuring consistency in drug calculations and visualizing cash flow within the institution. It fully meets the requirements for integrating pharmaceutical management with financial management.

SPD has evolved from solving procurement and distribution efficiency to digitalizing the whole process of purchasing, supplying, using and settling with digital traces, big data analysis and supervision, featuring digital intelligence. Any variety of abnormalities, price abnormalities, and dosage abnormalities can be pushed to the pharmacy regulator at the first time.

### 3.5 Criterion for evaluation

Drawing upon the perspectives on performance evaluation of public hospitals in China (Li et al., 2022) and relevant literature reports (George and Elrashid, 2023), we have identified a set of indicators for assessing the performance of this project, encompassing medical quality and safety, operational efficiency, and cost considerations. These indicators include:

#### 3.5.1 Drug loss reporting

Before the project implementation, medication expiration dates were solely reliant on manual inspection. However, with the introduction of this system, it automatically generates reminders for medications with a shelf life of less than 6 months. Each department submits monthly reports to the pharmacy warehouse regarding the quantity of damaged medications, which are then consolidated and analyzed by the pharmacy warehouse. The pharmacy warehouse maintains records of drugs including their names, quantities, and reasons for loss (such as expiration or damage). This indicator reflects issues related to drug maintenance, dispensing error rates, drug validity periods, and other aspects of drug quality and safety management.

#### 3.5.2 Inventory error

The inventory error was calculated by comparing the actual drug inventory with the expected amount, aiming for an ideal state of 0‰ error rate to indicate no discrepancies. The error rates for the 12 months prior to project implementation (February 2018 to January 2019) and the 12 months after project implementation (February 2019 to January 2020) were represented as medium and quartile range (P25, P75). This indicator assesses the level of financial management pertaining to hospital drugs.

#### 3.5.3 Cross-hospital drug collection volume

The total count of prescriptions for inter-campus medication retrieval is determined by tallying the instances where medication is prescribed at one campus and dispensed at another campus. For instance, if a patient receives a prescription at Campus A but retrieves the medication from Campus B, it indicates that Campus B acts as the dispensary on behalf of Campus A. Similarly, if a patient obtains a prescription at Campus B but collects the medication from Campus A, it signifies that Campus A serves as the dispensary on behalf of Campus B. This indicator reflects both the availability of drugs for patients as well as the degree of homogeneity in multi-hospital management practices.

#### 3.5.4 Drug cost (including economic cost and time cost)

The economic cost of medication refers to the proportion of hospital inventory. The sum of supplier inventory ratio and hospital inventory ratio amounts to 100%. Time cost encompasses the frequency of drug procurement (times/month), duration required for purchases (hours/month), accuracy of purchasing plan (%), drug delivery time (days/month), and drug inventory holding period (hours/month). This indicator captures both the financial burden faced by hospitals due to upfront payments as well as the operational efficiency associated with drug supply.

#### 3.5.5 Number of individuals served by cloud pharmacy services

The indicator pertains to the quantity of prescriptions obtained through online hospitals. This indicator highlights emerging models for drug supply along with comprehensive pharmaceutical care throughout all stages.

#### 3.5.6 Patient satisfaction

We distributed satisfaction questionnaires to patients via the hospital’s official WeChat account and short message service. The satisfaction levels in the questionnaire are categorized into five groups: satisfied, very satisfied, neutral, somewhat dissatisfied, and dissatisfied. The satisfaction rate is calculated as (the number of satisfied and very satisfied responses divided by the total number of responses) multiplied by 100%. Satisfaction represents the percentage of satisfactory and very satisfied responses among all returned questionnaires. The satisfaction of patients with pharmacies serves as an indicator that reflects their direct and personal experience of pharmaceutical care, thus playing a crucial role in assessing the quality of pharmaceutical care provided by hospitals.

### 3.6 Statistical analysis

The statistical analyses were conducted using SPSS software (version 22.0; IBM Corporation, Armonk, New York, United States). Normality tests were performed on the respective data before and after project implementation. For normally distributed data, descriptive statistics are presented as mean ± SD and analyzed using a *t*-test. Non-normally distributed data are described by median and interquartile range (P25, P75) and analyzed using a non-parametric test (Mann-Whitney U test). Statistical significance was defined as a two-tailed *p*-value less than 0.05. A *p*-value of <0.001 indicates highly significant differences.

## 4 Results

Statistical analysis is conducted on the changes in hospital pharmacy, emergency pharmacy, and supplier inventory before and after project implementation. Inventory data, cross-hospital medication retrieval data, supply efficiency, error rate, adverse reaction reporting quantity, and patient service status of internet cloud pharmacies are inventoried. The management level of pharmacy was improved, and the flow chart of continuous management improvement was shown in Supplementary Figure S3, and the key indicators were shown in Table 2.

**TABLE 2:** Improvement of drug management indicators before VMI project implementation (February 2017 to January 2019) and after VMI project implementation (February 2019 to January 2022).

Project	Before project implementation ( $\bar{X}$ ±SD)/(Min-MAX)	After project implementation ( $\bar{X}$ ±SD)/(Min-MAX)	*p*-value
Drug dispensing error (times/month)	5.0 ± 1.9/(3.0–8.0)	2.0 ± 1.1/(0–4.0)	<0.05
Number of expired varieties (pieces/month)	5.0 ± 1.7/(0–8.0)	1.0 ± 1.8/(0–2.0)	<0.05
Number of damaged drug varieties (boxes/month)	20.0 ± 7.3/(6.0–30.0)	5.0 ± 3.75 (0–14.0)	<0.05
Quantity of adverse reaction reports (cases/month)	1.0 ± 0.8/(0–2.0)	5.1 ± 1.6/(1.0–8.0)	<0.05

Reduce errors, reduce drug breakage rate, improve expiration date management, and increase the number of adverse reaction reports.

### 4.1 Reduce drug damage reporting

The SPD system’s purchase, sales and inventory reports and various data query functions can clearly reflect the accounting problems of drugs, from which you can find the procurement, application, allocation errors, and even the loss report caused by the adjustment errors. All kinds of errors can affect medication safety. Personnel can be penalized or educated/trained according to the type of problem identified while also making improvements to operational processes. Through these corrective measures, the prescription on dispensing errors were reduced from an average of more than 5.0 ± 1.9 cases per month to an average of 2.0 ± 1.1 cases per month (*p* < 0.05), which effectively reduced the prescription dispensing errors and further improved the drug safety.

The drug loss report reflects problems such as expired drugs, damaged drugs, incorrect allocation or drug losses caused by patient returns (Supplementary Figure S4). Expired and damaged drugs are common problems in the pharmaceutical supply chain. Therefore, strong policymakers and project support institutions must design an appropriate system to minimize them (Diriba et al., 2023). In terms of expiration date management, our hospital requires the inventory managers of suppliers to actively cooperate with pharmacists in effectively managing expiration dates and promptly resolving upcoming issues. Warehouse keepers are reminded to pay attention to the stacking height of easily broken medicines and handle them gently to reduce breakage. Through improvement, the amount of reported damage due to breakage of drugs is reduced. After the implementation of post-use settlement of drugs, the inventory belongs to the supplier, and the supplier and the hospital jointly bear the responsibility of monitoring the inventory. When the drug inventory of the supplier is about to expire, they will actively cooperate with the hospital to solve the problem of the expiration, so as to reduce the loss caused by the expiration of the drug, and the inventory error rate of our hospital has decreased. This collaborative approach enhances medication safety management in hospitals. After project implementation, the monthly average of expired varieties decreased significantly from 5.0 ± 1.7 pieces to 1.0 ± 1.8 pieces (*p* < 0.05). Additionally, the occurrence of damaged varieties reduced from 20.0 ± 7.3 boxes/month to 5.0 ± 3.75 boxes/month (*p* < 0.05). The hospital stipulates that returning drugs due to adverse reactions is a reasonable action, and any drug losses resulting from adverse reactions should be accompanied by the pharmacist responsible for returns filling out an adverse reaction report form. As a result, the number of adverse reaction reports has gradually increased from an average of 1.0 ± 0.8 per month to 5.1 ± 1.6 cases (*p* < 0.05), thereby strengthening the monitoring efforts on drug adverse reactions.

Through the analysis of the drug report, the corresponding rectification measures were implemented according to the causes of loss reporting, which improved the dispensing accuracy, reduced unnecessary loss reporting, and improved the management ability and service level of pharmaceutical personnel, as shown in Supplementary Figure S3. The process was continuously improved, and key indicators were shown in Table 2.

### 4.2 Inventory discrepancies have decreased

**FIGURE 2:**
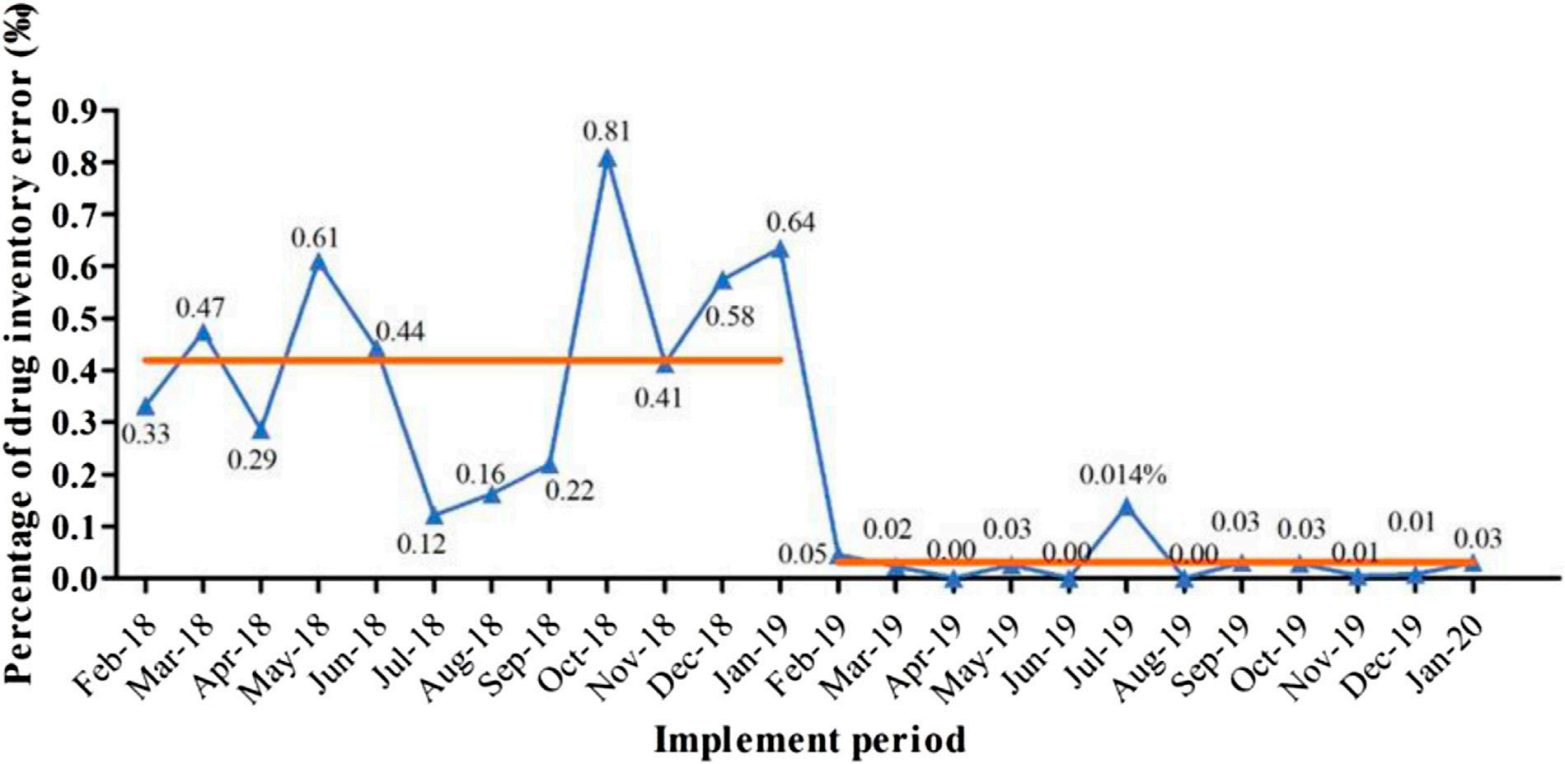
The percentage of drug inventory error in outpatient and emergency pharmacy before (2018/2-2019/1) and after project implementation (2019/2-2020/1). Note that the average monthly inventory error (shown as the orange line) reduced from 0.425‰ (0.240‰–0.600‰) to 0.025‰ (0.002‰–0.030‰) (*p* < 0.001) after the project execution.

Since the implementation of the settlement mode for drug consumption, daily sales information has been collected by the financial HIS system and SPD System, which is then uploaded to the OMS+VMI platform for reconciliation. This simplified process allows for timely issue resolution, ensuring daily account reconciliation and eliminating transmission errors in the information system. As a result, financial accounting can be synchronized, improving consistency between drug consumption amounts in the SPD system and prescription charges in hospitals. The implementation of the project resulted in a significant decrease in the monthly inventory error rate, from 0.425‰ (0.240‰–0.600‰) to 0.025‰ (0.002‰–0.030‰) (*p* < 0.001) (Figure 2).

### 4.3 Cross-campus medication pickup for patient convenience

The cross-hospital medication pickup model has formed a logistics management system for dispersed pharmaceuticals across multiple hospital areas, which poses significant challenges to the financial management of drug systems. After implementing this project, we restructured the electronic accounting data form for drugs so that they can be allocated and used in various hospital areas, synchronizing supply as much as possible for some scarce medications, and providing patients with higher accessibility and timeliness to drugs in different hospital regions (Supplementary Figure S5). Since the start of this project, it has served approximately 50,000 patients receiving treatment in different hospital campuses. This greatly improves patient convenience while ensuring seamless information flow. Patient satisfaction rates have significantly increased from 90.94 ± 2.10 (%) to 95.46 ± 1.90 (%) (*p* < 0.05). Table 3 shows the distribution of cross-campus medication pickups by patients between hospitals.

**TABLE 3:** The number of patients taking medicine across different campuses.

Year	Campus B dispenses drugs on behalf of campus A	Campus A dispenses drugs on behalf of campus B	In total
2019	2,198	226	2,424
2020	4,294	314	4,608
2021	12,989	187	13,176
2022	33,666	184	33,850
In total	53,147	911	54,058

### 4.4 The cost of hospital drugs has decreased

**FIGURE 3:**
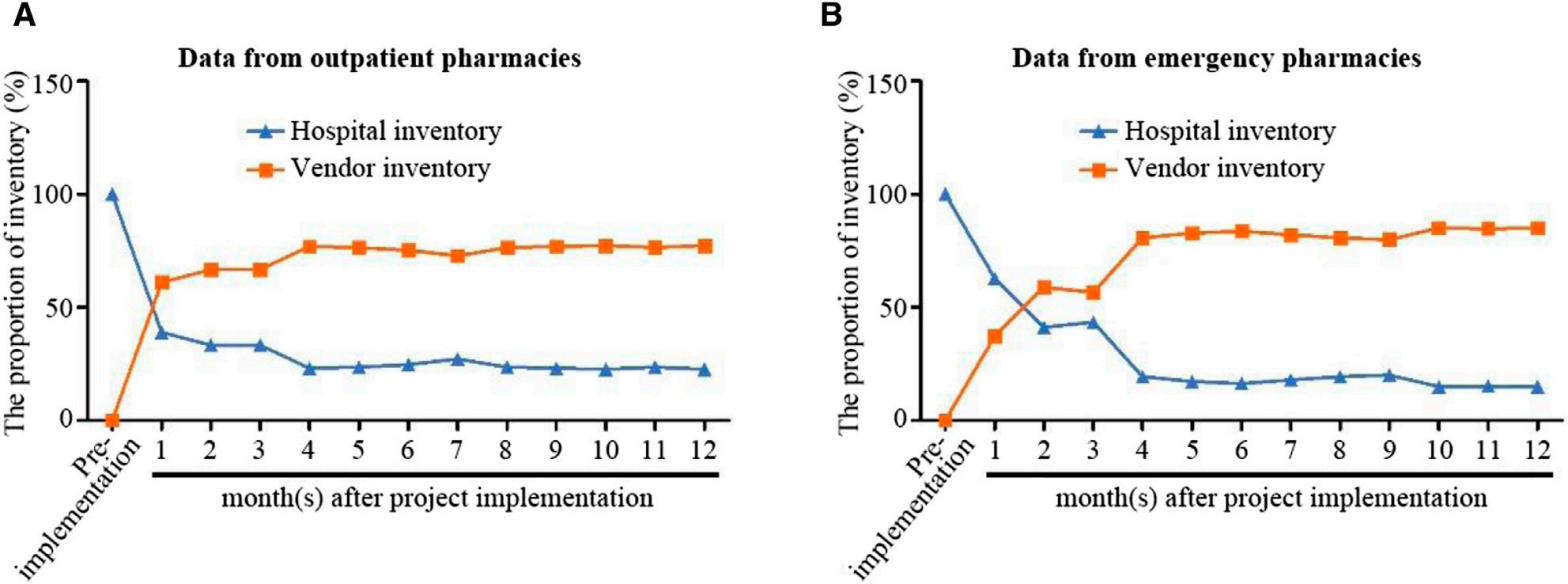
Changes in outpatient pharmacies **(A)** and emergency pharmacies **(B)** drug inventory before and after project implementation. The VMI project started in February 2019. Hospital inventory is represented by a blue line, while supplier inventory is represented by an orange line. **(A,B)** respectively indicate data from outpatient and emergency pharmacy.

The traditional settlement model requires hospitals to pay upfront when purchasing drugs. Prior to implementing VMI, the hospital had complete ownership of drug inventory, accounting for 100% of the stock. However, after project implementation and transfer of ownership to the supplier, the inventory of drugs in outpatient and emergency departments gradually reduced. As shown in Figure 3, 1 year after project implementation, hospital drug inventory has already decreased to around 20%. The use of post-consumption settlement mode is conducive to scientific management of drug inventory, accurate understanding of hospital/supplier inventory ratio, improvement of turnover rate, reduction of drug waste, simplification of reconciliation settlement process, and efficient utilization of hospital funds. It is worth noting that although drugs with special management requirements such as homemade preparations, narcotic drugs, Class I psychotropic drugs, and toxic drugs in hospitals are excluded from the post-consumption settlement model for policy reasons, the post-consumption settlement model is still effective in saving pre-drug funds, accounting for more than 50% of the savings.

For suppliers, real-time access to drug inventory data also prevents supply shortages or the accumulation of expired drugs caused by independent purchasing forecasts. This integrated approach to supply chain management also significantly reduces overall overheads for hospitals and suppliers.

In addition to reducing economic costs, the overall efficient intelligent hospital supply chain system improves the work efficiency of the operators in the warehouse and saves time costs. Table 4 shows a comparison of drug supply efficiency before and after project implementation.

**TABLE 4:** Comparison of the efficiency of drug supply before (February 2017 to January 2019) and after project implementation (February 2019 to January 2022).

Item	Before the project implementation ( $\bar{X}$ ±SD)/(Min-Max)	After the project implementation ( $\bar{X}$ ±SD)/(Min-Max)	Efficiency improvement (%)	*p*-value
Drug purchase frequency (Times/month)	39.9 ± 4.7/(30.0–48.0)	20.1 ± 2.2/(14.0–23.0)	49.6	<0.05
Time required for purchases (hours/month)	83.3 ± 7.0/(72.0–96.0)	20.7 ± 1.6/(17.0–23.0)	75.1	<0.05
Accuracy of purchasing plan (%)	88.1 ± 5.4/(80.0–96.0)	98.6 ± 1.1/(96.0–100.0)	11.9	<0.05
Drug delivery time (days/month)	20.0 ± 3.3/(10.5–24.0)	10.6 ± 4.1/(6.3–11.5)	47.0	<0.05
Drug inventory time (hours/month)	2.1 ± 0.7/(1.0–3.0)	1.0 ± 0.4/(0.5–2.0)	52.4	<0.05
Average of Efficiency improvement (%)			42.4	

### 4.5 Promote the development of cloud pharmacies

The establishment of a pharmaceutical supply chain provides a solid foundation for the construction of cloud pharmacies. By implementing an AI+VMI supply chain platform, most drug purchases can be settled after consumption, creating a cloud pharmacy model with large-scale warehouse distribution. With the emergence of internet hospitals, doctors can issue prescriptions online, and hospital pharmacists can review them electronically. Patients have the option to collect their medication from the hospital or utilize the settlement model provided by large warehouses, where suppliers directly deliver the drugs to their doorstep (Bu et al., 2022). Our hospital’s inventory report for cloud pharmacies clearly displays information on allocation, distribution, and settlement of these drugs across different campuses. This establishes a digital foundation for innovative technologies such as prescription collection codes during epidemic periods.

Since its establishment 3 years ago, our Internet cloud pharmacy has distributed over 360,000 online prescriptions, with a peak of over 1,000 prescriptions per day. Considering the transportation and time costs for each patient, each online prescription can save patients at least 200 yuan. Our Internet cloud pharmacy has saved a total cost of over 70 million yuan for patients, ranking first in online treatment for ophthalmology and otolaryngology nationwide. Artificial Intelligence (AI) and Big Data Analytics (BDA) can guarantee accuracy within the range of task parameters (Alahmari et al., 2022) and have the potential to significantly improve resilience of supply chains (Zamani et al., 2022). The cloud pharmacy greatly facilitated patients’ online follow-up visits and medication dispensing while reducing the risks associated with personnel gathering and cross-infection.

## 5 Discussion

With the advent of the big data era, AI has been widely applied in the business environment; however, its implementation is still in its early stages or not properly executed. Proper implementation can yield significant positive results and transform the supply chain (Riahi et al., 2021). The adoption of AI in supply chain management can enhance performance, reduce costs, minimize losses, and make the supply chain more agile and responsive (Riahi et al., 2021). AI helps supply chain companies strengthen their analytical capabilities, thereby improving operational performance (Dubey et al., 2021) and financial management efficiency (Akhtar et al., 2022). Compared to other industries, healthcare institutions have relatively complex supply chains that require the implementation and utilization of AI to achieve intelligent supply chains and deliver high-quality medical services at lower costs (Bialas et al., 2023).

### 5.1 Advantages of intelligent supply chain management

The integration of AI into our supply chain has transformed the decision-making process from relying solely on machine input in the past to now having machines drive decisions under human supervision. Our system utilizes AI-based VMI intelligent reconciliation to settle drug consumption, effectively reducing hospital inventory and manpower costs. By allowing suppliers real-time access to drug inventory data, it prevents supply shortages or accumulation of expired drugs caused by independent procurement forecasts. This comprehensive approach to supply chain management significantly reduces overall management expenses for both hospitals and suppliers.

The various functions implemented by the SPD system and the various query reports generated can reflect every detail of pharmacy management. The automatic push function of abnormal data can enable managers to find problems in time and make targeted continuous improvements, thus improving the management ability and service level of pharmaceutical personnel.

### 5.2 The difficulty in inventory management after the settlement of drug consumption

#### 5.2.1 Support from information systems is crucial

To effectively manage the inventory of drugs after consumption settlement, it is necessary to establish a robust hospital intelligent supply chain system. The SPD system seamlessly integrates with multiple internal information systems within the hospital, enabling seamless data exchange across different platforms. In cases where drugs are transferred to the hospital and require post-consumption settlement, there is often a delay in invoicing, resulting in combinations of multiple medications on a single invoice. To verify invoices corresponding to specific batched drugs, it is necessary to search through various platforms such as SPD and VMI for validation. However, this process may lead to potential data issues such as medication errors or inventory discrepancies, which can result in significant systemic errors. Furthermore, when sharing sensitive hospital data with suppliers, strict control over access permissions and enhanced supervision measures are essential to ensure the security and integrity of core system data (Qian et al., 2023). In order to ensure robust data security, our hospital has implemented a user-based permission system, ensuring data security through traceable operation records.

#### 5.2.2 Multi-departmental collaboration

The entire system involves suppliers, pharmacy departments, and finance departments. To avoid monopolies, it is advisable to select two or more strong suppliers with good reputation and strong cooperation capabilities to jointly carry out SPD services, implementing a multi-supplier supply model that assesses service quality from multiple dimensions such as timely delivery rate, stockout rate, and service evaluation. In order to comply with drug management requirements and financial regulations within the hospital (Gorbenko et al., 2023), careful consideration must be given to the design of the business system. Therefore, the implementation of the business system requires support from various departments within the hospital and continuous optimization of business processes. Under the guidance of hospital management department, active cooperation and support from various departments ensure smooth completion of the project.

#### 5.2.3 Further policy support is needed

When implementing AI+VMI to optimize the supply chain, hospitals must also consider government policies such as the national centralized drug procurement policy (Bas et al., 2023). This policy will affect the invoicing process after consumption settlement, including issues like drug repricing, changes in drug medical insurance codes, and price reductions. In order to comply with the requirements of the “Shanghai Medical Procurement Service and Supervision Information System” or medical insurance policies, hospitals need to take corresponding measures regarding changes in drug procurement and settlement rules. For example, before pharmaceutical purchases become invalid, timely conversion of supplier inventory into hospital inventory for invoice issuance; returning products to suppliers based on batch numbers through reverse warehousing; strengthening dosage monitoring for drugs with changed medical insurance settlement rules during the buffer period for settlement to prevent expiration-related unsettled issues and potential losses for both hospitals and suppliers.

The application of AI+VMI provides convenience for post-consumption settlements of most drugs. However, due to local policies’ influence, some drugs still require traditional practices involving paper invoices alongside goods delivery notes which pose challenges towards achieving zero inventory management. Successful implementation of post-consumption settlements requires continuous policy support while establishing top-level design and comprehensive regulations.

### 5.3 Limitations and future work

The data samples in this study were exclusively obtained from a tertiary specialist hospital, characterized by a fixed range of drugs and limited quantity, which may limit the generalizability to the practices of more diverse clinical drug settings in general hospitals. Moreover, the evaluation indicators established might not encompass all relevant aspects; only the impact of improvement indicators on downstream hospital pharmacy departments as part of the supply chain system was analyzed, with less consideration given to their influence on the entire supply chain. In the field of supply chain research, some scholars have proposed that the application of artificial intelligence (AI) technology in supply chains is meaningful only when the error term falls within specific intervals (Gao et al., 2023). Currently, AI functions are still limited to single tasks. Although our hospital has implemented an AI+VMI supply chain system to automate procurement plan generation, low stock warnings, and reconciliation processes, thereby enhancing pharmaceutical service capabilities, the system’s functionality cannot entirely replace manual labor. For instance, due to frequent drug replacements and other factors, while the current inventory status can indicate high and low reserves after automatic purchase plan generation, it still requires manual review and adjustment of purchase varieties. Additionally, achieving automated and accurate demand prediction remains unattainable. For instance, reconciliation failure occurs due to drug price adjustments, necessitating manual intervention for the reconciliation of pharmaceutical invoices.

In the future, our institute will continue to conduct in-depth research and innovation on the supply chain system with the aim of achieving real-time monitoring and efficient, transparent whole-process inventory management through the development of a visual multi-department inventory and consumption system. Additionally, we will perform multidimensional refined data analysis including cost analysis and key variety analysis. This will enhance prompt functions for alternative drugs when shortages occur, strengthen precise decision-making through digital big data analysis, achieve integrated development of online and offline channels along with in-hospital and out-of-hospital parties’ integration, ultimately creating an interconnected high-quality supply chain digital intelligence ecosystem.

## 6 Conclusion

In summary, the integration of AI and VMI in the pharmaceutical supply chain optimizes management through the use of information technology and modern logistics concepts. By implementing inventory management models, it effectively addresses a significant portion of procurement costs for hospitals, aligning with the current trends in healthcare reform. The primary objectives are to reduce hospital expenses, enhance scientific and transparent drug management, improve work efficiency, minimize errors and wastage, while ensuring reasonable and stable medication stock levels for hospitals to ensure accessibility for patients. This mutually beneficial approach fosters collaboration between hospitals and suppliers.

## Data Availability

The original contributions presented in the study are included in the article/Supplementary Material, further inquiries can be directed to the corresponding authors.
