# Supplementary material for: Management of drug supply chain information based on “artificial intelligence + vendor managed inventory” in China: perspective based on a case study
Source: Front Pharmacol. 2024 Jul 16;15:1373642. doi: 10.3389/fphar.2024.1373642 (PMC11286579; doi:10.3389/fphar.2024.1373642)
Supplement: Supplementary file 1 [file DataSheet1.PDF]

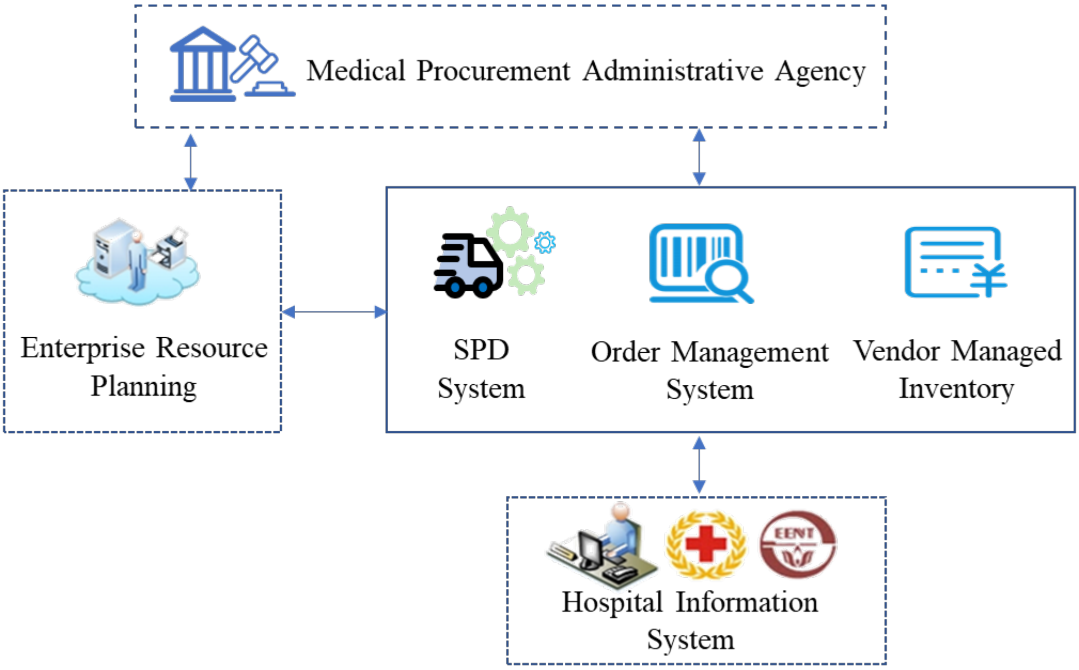

Supplementary Figure 1| Framework layout of the post-consumption settlement system for pharmaceuticals.

Under the supervision of government healthcare procurement management agencies, the supply chain platform facilitates synchronous integration between hospitals and multiple suppliers. It is typically deployed on cloud or public networks, enabling access for both hospitals and suppliers.

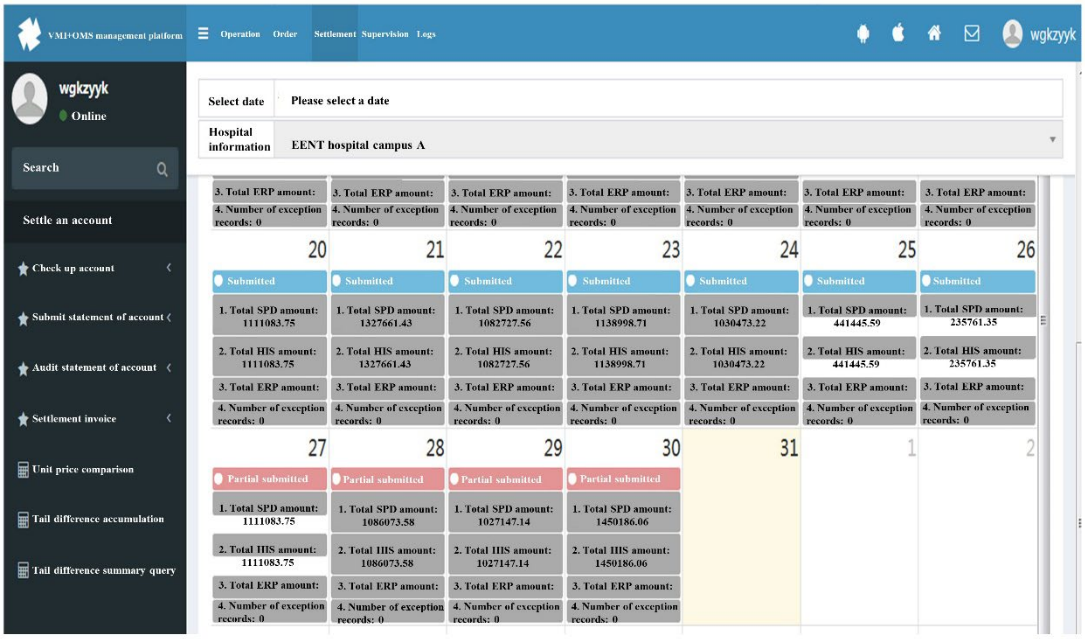

Supplementary Figure 2| The interface of VMI reconciliation (Exemplified by the April 2023 report)

The reconciliation results between SPD and HIS are displayed on the main screen, with any anomalous data pertaining to amount, quantity, unit price or owner being highlighted in red within the list.

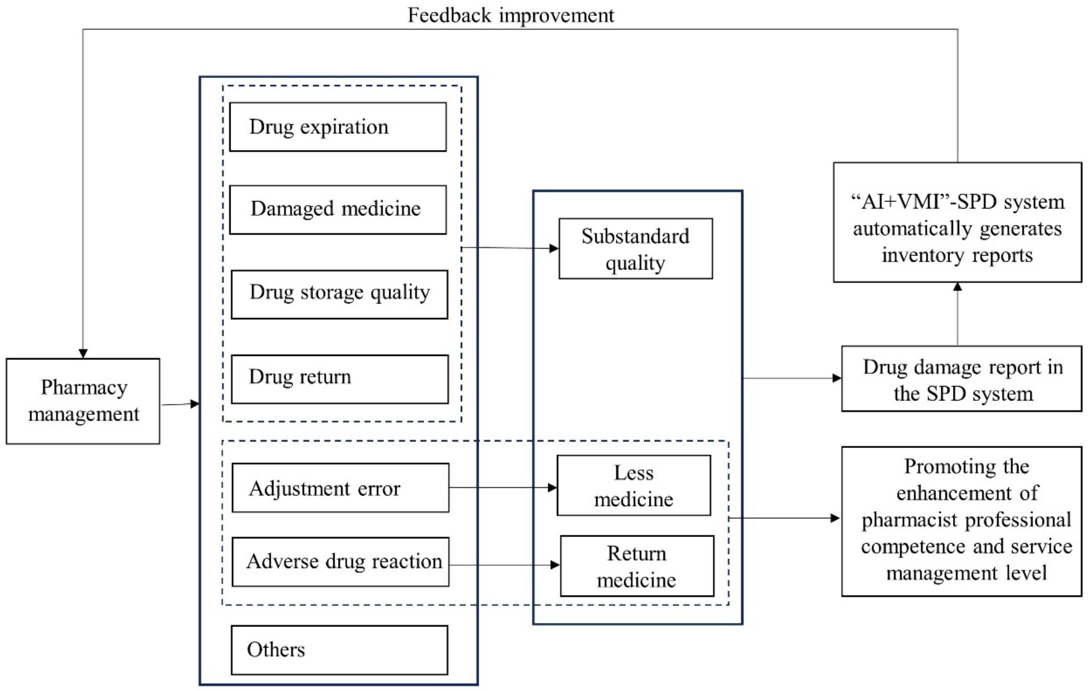

Supplementary Figure 3| Continuous improvement flowchart for medicines management

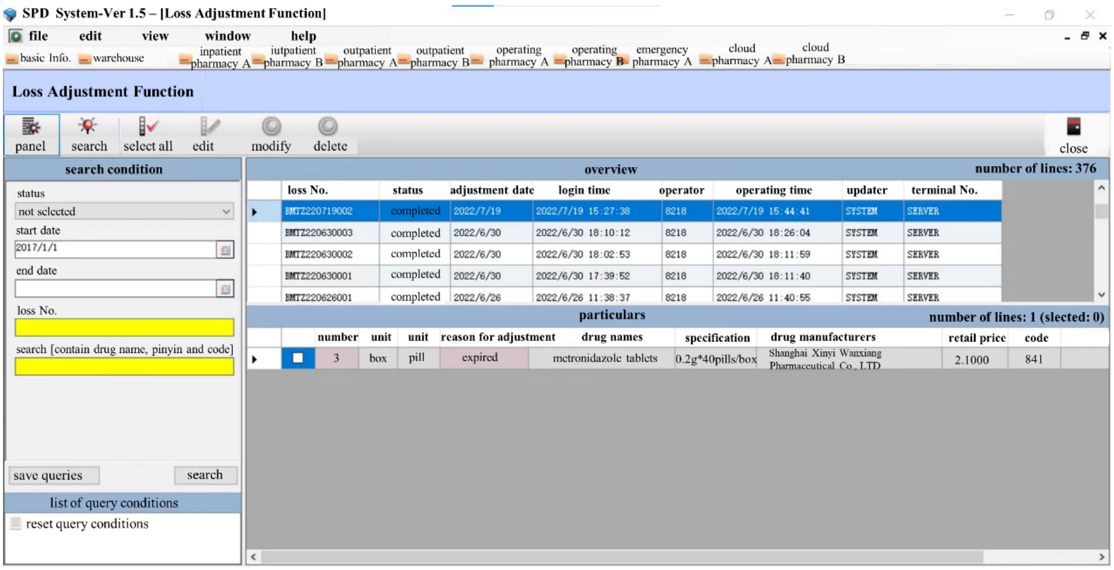

Supplementary Figure 4| Loss adjustment function

The reason for reporting losses of medicines should be provided.

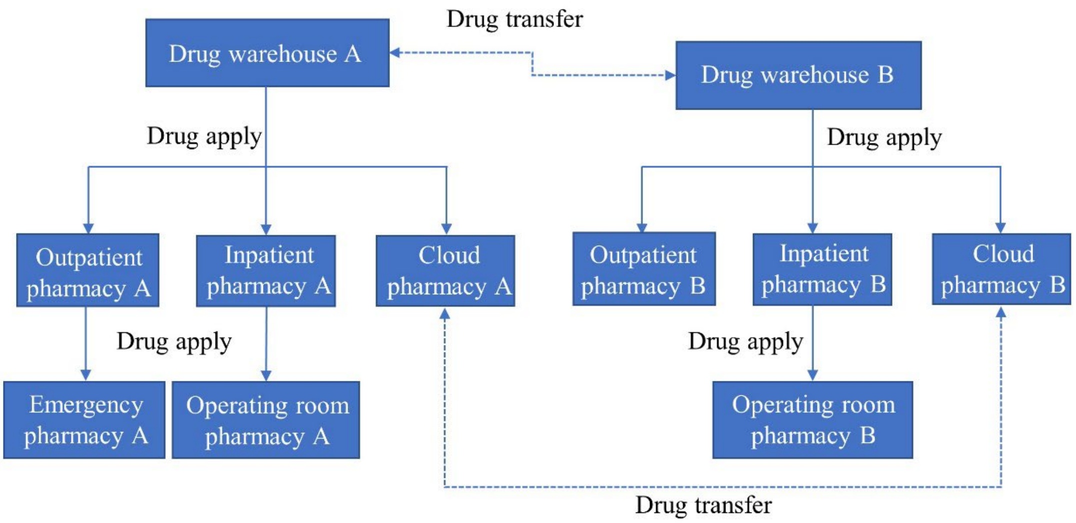

Supplementary Figure 5| Process for drug application and transfer.

Note: Lower-level pharmacies can only request drugs from higher-level pharmacies within the same campus, and internal redistribution is allowed within the same campus. Additionally, inter-campus drug warehouses are permitted to transfer drugs between each other."

Supplementary Table 1| The chart of purchase-sales-stock Statement

Categories	Items	Purchase Price of western medicine (CNY)	Purchase price of Chinese patent medicine (CNY)	Total purchase price (CNY)
The Beginning of the period		916464.79	1224433.49	2140898.28
Input	Departmental transfer	11744.83	61070.90	72815.73
	Departmental application	910602.05	2590480 15	3501082.14
	Drug Inventory surplus	2478.00	2649.70	5127.70
	Outpatient pharmacy return	8897.61	3680.58	12578.19
	medicines transferred from the outpatient pharmacy B to pharmacies in Campus A	9815 28.50	142.20	981670.70
Total amount for input		1915250.99	2658023.47	4573274.46
Output	Departmental drug returns	965.00	14919.20	15884.20
	Departmental transfer	34258.66	184809.93	219068.59
	Reporting of drug losses	877.07	470.16	1347.23
	Outpatient pharmacy dispensing	1635516.00	2670966.26	4306482.34
	medicines transferred from the pharmacies in Campus A to outpatient pharmacy B	59570.33	0.00	59570.33

Total amount for output	1731187.14	2871165.55	4602352.69
The ending of the period	1100528.64	1011291.41	2111820.05

Supplementary Table 2| Intelligent account reconciliation of drugs between Campus

Inventory of medicines in Campus A		Inventory of medicines in Campus B	
External transfer from warehouse B to warehouse A (hospital cargo owner)	11736.84	External transfer from warehouse A to warehouse B.(hospital cargo owner)	115149.60
External transfer from outpatient pharmacy B to the warehouse A	617312.31	External transfer from outpatient pharmacy A to the warehouse B	104548.42
external transfer from outpatient pharmacy B to Cloud pharmacy A (hospital cargo owner)	132826.15		
Total	761875.30	Total	219698.02
Warehouse exit from Campus A		Warehouse exit from Campus B	
medicines transferred from warehouse A to warehouse B (Hospital inventory)	106174.60	medicines transferred from warehouse B to warehouse A	9240.20
medicines transferred from any of the pharmacies in Campus A to the outpatient pharmacy in Campus B	58415.82	medicines transferred from any of the pharmacies in Campus B to the outpatient pharmacy in Campus A	429512.89
medicines transferred from warehouse A to warehouse B (Supplier Inventory)	55107.60	medicines transferred from warehouse B to warehouse A （Supplier Inventory）	323122.21
Total	219698.02	Total	761875.30
